# Ex Vivo Antiplatelet Effects of Oral Anticoagulants

**DOI:** 10.3390/jcdd11040111

**Published:** 2024-03-31

**Authors:** Giulia Renda, Valentina Bucciarelli, Giulia Barbieri, Paola Lanuti, Martina Berteotti, Gelsomina Malatesta, Francesca Cesari, Tanya Salvatore, Betti Giusti, Anna Maria Gori, Rossella Marcucci, Raffaele De Caterina

**Affiliations:** 1 Department of Neuroscience, Imaging and Clinical Sciences and Center for Advanced Studies and Technology, G. d’Annunzio University Chieti-Pescara, 66100 Chieti, Italy; tanyasalvatore87@gmail.com; 2 Cardiovascular Sciences Department, Azienda Ospedaliero-Universitaria delle Marche, 60121 Ancona, Italy; valentina_bucciarelli@yahoo.it; 3 Department of Experimental and Clinical Medicine, University of Florence, 50121 Florence, Italy; giulia.barbieri@unifi.it (G.B.); martina.berteotti@unifi.it (M.B.); cesarif@aou-careggi.toscana.it (F.C.); betti.giusti@unifi.it (B.G.); annamaria.gori@unifi.it (A.M.G.); rossella.marcucci@unifi.it (R.M.); 4 Department of Medicine and Aging Sciences and Center for Advanced Studies and Technology, G. d’Annunzio University Chieti-Pescara, 66100 Chieti, Italy; paola.lanuti@unich.it; 5 Cardiology Unit, National Institute of Health and Science on Aging (INRCA), 64125 Ancona, Italy; g.malatesta@inrca.it; 6 Cardiology Division 1-Pisa University Hospital, University of Pisa, 56124 Pisa, Italy; raffaele.decaterina@unipi.it; 7 Fondazione Villa Serena per la Ricerca, 37011 Città Sant’Angelo, Italy

**Keywords:** oral anticoagulants, non-vitamin K antagonist oral anticoagulants, NOACs, direct oral anticoagulants, DOACs, platelets, antiplatelet effects

## Abstract

Background: The impact of non-vitamin K antagonist oral anticoagulants (NOACs) on platelet function is still unclear. We conducted a comprehensive ex vivo study aimed at assessing the effect of the four currently marketed NOACs on platelet function. Methods: We incubated blood samples from healthy donors with concentrations of NOACs (50, 150 and 250 ng/mL), in the range of those achieved in the plasma of patients during therapy. We evaluated generation of thrombin; light transmittance platelet aggregation (LTA) in response to adenosine diphosphate (ADP), thrombin receptor-activating peptide (TRAP), human γ-thrombin (THR) and tissue factor (TF); generation of thromboxane (TX)B_2_; and expression of protease-activated receptor (PAR)-1 and P-selectin on the platelet surface. Results: All NOACs concentration-dependently reduced thrombin generation compared with control. THR-induced LTA was suppressed by the addition of dabigatran at any concentration, while TF-induced LTA was reduced by factor-Xa inhibitors. ADP- and TRAP-induced LTA was not modified by NOACs. TXB_2_ generation was reduced by all NOACs, particularly at the highest concentrations. We found a concentration-dependent increase in PAR-1 expression after incubation with dabigatran, mainly at the highest concentrations, but not with FXa inhibitors; P-selectin expression was not changed by any drugs. Conclusions: Treatment with the NOACs is associated with measurable ex vivo changes in platelet function, arguing for antiplatelet effects beyond the well-known anticoagulant activities of these drugs. There are differences, however, among the NOACs, especially between dabigatran and the FXa inhibitors.

## 1. Introduction

All anticoagulants are expected to have some indirect effects on platelet function because they interfere with the generation or activity of thrombin, which is a potent platelet agonist [1]. Interference of anticoagulants with platelet function can explain part of their antithrombotic efficacy on the one hand, and additive or synergistic effects with antiplatelet agents on the other.

Previous studies showed that anticoagulants (direct or indirect thrombin inhibitors) reduce markers of thrombin generation and, concomitantly, thrombin-induced platelet activation [2,3]. There are, however, very few data about antiplatelet effects of non-vitamin K antagonist oral anticoagulants (NOACs). Both the direct thrombin inhibitor dabigatran—by inhibiting thrombin activity—and the direct factor (F) Xa inhibitors rivaroxaban and apixaban, indirectly preventing thrombin production, have been shown to inhibit thrombin-mediated effects and reduce the endogenous thrombin potential (ETP) in a concentration-dependent manner [4,5,6,7]. Recent studies have also shown that both dabigatran and FXa inhibitors affect platelet aggregation induced by thrombin [8,9] but also by tissue factor (TF) [10,11], which indirectly stimulates platelet aggregation through thrombin generation via the extrinsic coagulation pathway [12,13]. Moreover, dabigatran inhibited thrombin-induced platelet activation and aggregation in a concentration-dependent fashion [14], but also appeared to reduce platelet aggregation induced by thrombin receptor-activating protein (TRAP) [15], while arachidonic acid- and adenosine diphosphate (ADP)-induced platelet reactivity was not influenced [16]. In vitro studies have also demonstrated that dabigatran induces an overexpression of protease-activated receptor-1 (PAR-1) on the platelet surface [17]. A recent study conducted in patients with atrial fibrillation has confirmed that dabigatran treatment increases thrombin receptor density on the platelet surface, enhancing platelet reactivity [18]. Platelet aggregation induced by arachidonic acid, ADP, collagen and epinephrine did not appear to be affected, conversely, in patients taking apixaban and rivaroxaban, while TRAP-induced aggregation was reduced [19,20,21]. As these studies with various anticoagulants have been conducted in disparate experimental and clinical conditions, often in patients chronically treated with aspirin and—occasionally—with inhibitors of glycoprotein (GP) IIb/IIIa, all drugs that themselves affect platelet aggregation, the real impact of NOACs on platelet function has not yet been fully clarified and compared. 

We therefore conducted a comprehensive ex vivo study aimed at assessing the effect of the four currently marketed NOACs—the thrombin inhibitor dabigatran and the FXa inhibitors rivaroxaban, apixaban and edoxaban—on platelet function. In vivo comparisons of the effects of NOACs on platelet function will be the subject of a complementary report.

## 2. Methods

This study was conducted at the Experimental Cardiology Unit and at the flow cytometry facility of the Center for Advanced Studies and Technology (CAST) at the G. d’Annunzio University of Chieti-Pescara, and at the Atherothrombotic Disease Unit, Azienda Ospedaliero-Universitaria Careggi, University of Florence, Italy. The Center of Chieti was involved with the experiments with dabigatran, rivaroxaban and apixaban, the Center of Florence with the experiments with edoxaban. Experimental methods were shared by the two centers and inter-reproducibility of the findings cross-checked.

The ex vivo experiments of this study consisted of incubation of blood samples from healthy volunteers with varying concentrations of the NOACs. For this purpose, we enrolled 20 healthy volunteers in Chieti for dabigatran, apixaban and rivaroxaban experiments, and other 20 volunteers in Florence for edoxaban experiments. The two populations, consisting of 17 males and 23 females, had overlapping characteristics, with a median age of 30 (interquartile range 27–42), a median BMI of 22 (interquartile range 21–26), and with blood group 0^+^ in 38%, A^+^ in 33%, B^+^ in 17%, 0^−^ in 8%, and AB^+^ in 4% of volunteers. They were also asked to abstain from non-steroidal anti-inflammatory drugs (NSAIDs) the week before study, allowing paracetamol as the only antipyretic/analgesic drug in case of need. We evaluated the generation of thrombin, platelet aggregation, generation of thromboxane (TX)B_2_, and expression of PAR-1 (the main platelet thrombin receptor) and of the marker of platelet activation P-selectin on the platelet surface. Anticoagulants were tested at increasing concentrations of 50, 150 and 250 ng/mL, in the range of those measured in the plasma of patients during chronic therapy [22,23,24,25], and after preliminary testing of a wider range of intermediate concentrations from 30 to 300 ng/mL (30, 50, 75, 100, 150, 200, 250 and 300 ng/mL) in the initial experimental setups. In order to enable direct comparisons between molecules with different molecular weights, we also report 50, 150 and 250 ng/mL concentrations of each NOAC in molar units: dabigatran (79.66, 238.97 and 398.28 nmol/L, respectively), apixaban (108.81, 326.44 and 544.07 nmol/L, respectively), rivaroxaban (114.71, 344.13 and 573.55 nmol/L, respectively) and edoxaban (91.23, 273.69 and 456.16 nmol/L, respectively).

Dabigatran, rivaroxaban, apixaban and edoxaban pure substances were provided by Boehringer Ingelheim (Ingelheim am Rhein, Germany), Bayer AG (Wuppertal, Germany), Bristol Myers Squibb Company (New Brunswick, NJ, USA) and Daiichi Sankyo (Chūō, Tokyo, Japan). Drug powders were reconstituted with dimethylsulfoxide (DMSO) to form stock solutions according to manufacturers’ recommendations for non-clinical investigations [12,26,27,28] and stored at −80 °C before use.

The study protocol was conducted in accordance with the principles of the Declaration of Helsinki and approved by the Institutional Ethics Committees at both the Universities of Chieti and Florence. Written informed consent was obtained from all study participants.

### 2.1. Blood Samples

Antecubital venous blood was drawn using a 21 G needle and collected in 3.2% (*vol*:*vol*) citrated test tubes for the assessment of the endogenous thrombin potential, and for platelet aggregation and flow cytometry analyses; glass serum test tubes were used for the TX generation test. 

### 2.2. Calibrated Automated Measurement of Thrombin Generation (CAT)

Citrated whole blood was centrifuged at 2000× *g* for 20 min (min) at 0 °C to obtain platelet-poor plasma (PPP); samples were then stored at −80 °C until further processing. The day of the experiment, increasing concentrations of the four NOACs (50, 150, 250 ng/mL), or vehicle without drug, were added to platelet-poor plasma (PPP, see below), and incubated for 15 min at room temperature. 

Thrombin generation—as a global measure of the whole clotting system’s capacity—was assessed according to the method described by Hemker et al. [29], with a calibrated automated thrombogram (CAT) using a Fluoroskan Ascent^®^ microplate fluorimeter (Thermo Fisher Scientific, Waltham, MA, USA). Fluorescence intensity was detected at wavelengths of 390 nm (excitation filter) and 460 nm (emission filter). For this purpose, 80 μL of PPP was dispensed into the wells of round-bottomed 96-well microtiter plates. Next, 20 μL of a PPP reagent containing 5 pM tissue factor/4 μM phospholipids (Diagnostica Stàgo, Asnières sur Seine Cedex, France) was added to the PPP samples. The starting reagent (20 μL per well) contained the fluorogenic substrate (amino-methyl-coumarin, AMC) and CaCl_2_. A Thrombinoscope^®^ software (version 3.0.0.26, Thrombinoscope BV, Maastricht, The Netherlands) enabled the calculation of thrombin activity against the calibrator, and displayed thrombin activity against time. All tests were carried out in duplicate, and measurements were completed within 90 min. Parameters derived from the CAT were the endogenous thrombin potential (ETP, corresponding to the area under the CAT curve, reflects the total amount of generated thrombin, and is expressed as nM × min), the peak height of thrombin generation (corresponding to the maximum amount of thrombin that can be generated by the plasma sample during the thrombin burst, and recorded in nM), time to peak (representing the time course of the thrombin generation curve up to the formation of the thrombin peak height, in min) and the lag time before the curve take-off (in min). 

### 2.3. Platelet Aggregation Studies

Citrated plasma was immediately obtained from citrated whole blood (WB) with a two-step centrifugation. For the first centrifugation, WB was centrifuged at 200× *g* for 15 min to obtain platelet rich-plasma (PRP) as the supernatant, and this was then transferred to an empty polypropylene sterile tube. The remaining WB underwent a second centrifugation round at 2000× *g* for 20 min to obtain PPP. Platelet count in the PRP was checked by means of a Multisizer 3 Coulter Counter (Beckman Coulter, Brea, CA, USA) and used to dilute PRP with PPP if platelet count was >350.000/μL.

Increasing concentrations of the four NOACs (50, 150, 250 ng/mL), or their vehicle (dimethylsulfoxide, DMSO) without drug, were added to PRP and incubated for 15 min at room temperature before performing aggregometry studies. 

Light transmittance aggregometry (LTA) was evaluated according to Born’s method [30]. Platelet aggregation induced by adenosine diphosphate (ADP, 5 µM, Mascia Brunelli, Milano, Italy), human γ-thrombin (THR, 1.5 ng/mL, Enzyme Research Laboratories, South Bend, IN, USA), thrombin receptor-activating peptide (TRAP, 10 μM, Sigma Aldrich, St. Louis, MO, USA) and tissue factor (TF Innovin, diluted 1:1.000, Siemens, Berlino, Germany) was then measured in PRP using a light transmittance platelet aggregometer (Chrono-Log, Havertown, PA, USA). Human γ-thrombin was used because it is a non-coagulant form of thrombin that retains most of its platelet-activating capacity [31].

### 2.4. Thromboxane Generation

Increasing concentrations of the four NOACs (50, 150, 250 ng/mL), or their vehicle as control, were added to 1 mL whole blood in glass tubes and incubated for 60 min at 37 °C in order to evaluate TXB_2_ production as a platelet reactivity biomarker in the presence of NOACs, in comparison to samples incubated with the vehicle representing the physiological situation. Samples were then centrifuged at 2000× *g* at 0 °C for 15 min and the upper layer collected and stored at −20 °C until analysis. Serum thromboxane (TX) B_2_ was measured in duplicate using a TXB_2_ enzyme immunoassay (EIA) kit (Cayman Chemical, Ann Arbor, MI, USA) according to the manufacturer’s instructions. 

### 2.5. Flow Cytometry

Increasing concentrations of the four NOACs (50, 150, 250 ng/mL), or vehicle without drug, were added to citrated whole blood and incubated for 15 min at room temperature. Then, samples were incubated with fluorescent antibodies for CD41 [A-Human CD41a allophycocyanin (APC), Becton Dickinson Biosciences, La Jolla, CA, USA], P-selectin (Becton Dickinson) and PAR-1 expressed on the platelet surface. Due to the different periods of the study, we used an indirect PAR-1 antibody (Zenon Alexa Fluor 488 Mouse Ig, Histoline Laboratories, Milano, Italy) for the experiments conducted with dabigatran, and a direct PAR-1 antibody (Human PAR 1 Alexa Fluor 488 MAb, R&D, Minneapolis, MN, USA) for the experiments with rivaroxaban, apixaban and edoxaban. In a subset of samples (*n* = 5), the two above-mentioned anti-PAR1 antibodies were compared to ascertain their overlapping ability to stain the samples with respect to the related control samples, and the signal-to-noise ratio was evaluated. Antibodies were added to 100 μL of whole blood. After incubation for 30 min at room temperature in the dark, 1 mL of an erythrocyte-lysing solution (FACS Lysing Solution, Becton Diickinson, Franklin Lakes, NJ, USA) was added to each sample and incubated 5 min at room temperature. We acquired 1 × 10^6^ events/sample by flow cytometry (FACSVerse, BD Biosciences—three laser-, eight color-configuration). Each antibody/reagent was titrated (8-point titration) under the assay’s conditions. Reagent titrations were calculated according to current guidelines [32].

Instrument performances, data reproducibility and fluorescence calibrations were consistent throughout the study period and between the two laboratories involved in this study, as checked by the Cytometer Setup & Tracking Module, and further validated by the acquisition of Spherotech 8 peack Rainbow Beads (Becton Dickinson). In order to evaluate non-specific fluorescence, Fluorescence Minus One (FMO) controls were used. Compensation was assessed using CompBeads (Becton Dickinson), as recommended by recent guidelines [32], and single-stained fluorescent samples. Data were analyzed using FACSDiva v 6.1.3 (Becton Dickinson) and FACSuite v. 1.0.5 (Becton Dickinson) with FlowJo v. 8.8.6 (Becton Dickinson). Concentrations of cell suspensions were obtained by volumetric count. Data were reported as mean fluorescence intensity (MFI) ratios. 

### 2.6. Statistical Analyses

Results were expressed as median and interquartile range (IQR). To test for differences among groups, we used the one-way ANOVA for repeated measures with Tukey’s multiple comparison test for normally distributed variables. For data with a non-normal distribution, we used the non-parametric Kruskal–Wallis test with Dunn’s test for multiple comparisons. 

## 3. Results

### 3.1. Calibrated Automated Measurement of Thrombin Generation

Thrombin generation parameters are reported in Table 1. All NOACs reduced thrombin generation compared with controls in a concentration-dependent manner, although with some differences between drugs in the measured parameters. 

**Figure 1:**
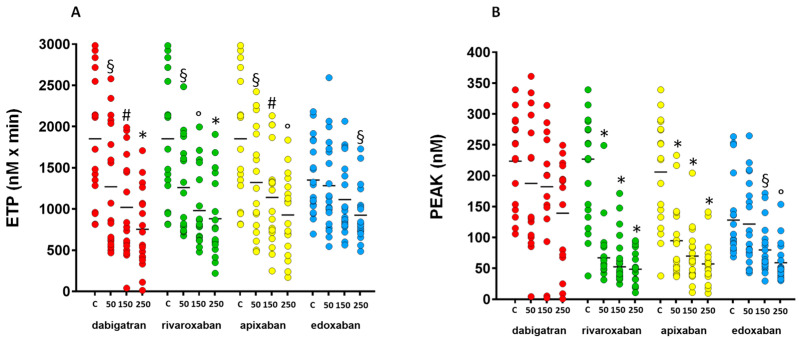
Thrombin generation parameters after the addition of increasing concentrations of dabigatran, rivaroxaban, apixaban and edoxaban. Dot plots report all single values of endogenous thrombin potential (ETP, panel (**A**)) and peak of thrombin generation (panel (**B**)). The horizontal lines represent the mean values. * *p* < 0.0001, ° *p* < 0.001, ^#^ *p* < 0.01, ^§^ *p* < 0.05 vs. the corresponding controls. C = control; 50 = 50 ng/mL; 150 = 150 ng/mL; 250 = 250 ng/mL.

Dabigatran significantly reduced ETP at all tested concentrations, (*p* < 0.05 at 50 ng/mL, *p* < 0.01 at 150 ng/mL, *p* < 0.0001 for 250 ng/mL vs. control) (Figure 1A), but did not significantly affect peak of thrombin generation, time to peak or lag time. 

Rivaroxaban and apixaban modified all thrombin generation parameters in a concentration-dependent manner. Rivaroxaban significantly reduced ETP (*p* < 0.05 at 50 ng/mL, *p* < 0.001 at 150 ng/mL, *p* < 0.0001 at 250 ng/mL vs. control) (Figure 1A), peak of thrombin generation (*p* < 0.0001 at all concentrations vs. control) (Figure 1B), time to peak (*p* < 0.001 at 50 ng/mL, *p* < 0.0001 at 150 ng/mL and 250 ng/mL vs. control) (Figure 1, panel C) and lag time (*p* < 0.05 at 50 ng/mL, *p* < 0.0001 at 150 ng/mL and 250 ng/mL vs. control). Apixaban significantly reduced ETP at concentrations of 150 ng/mL (*p* < 0.01 vs. control) and 250 ng/mL (*p* < 0.001 vs. control) (Figure 1A), and at all concentrations reduced peak of thrombin generation (*p* < 0.0001 at all concentrations vs. control) (Figure 1B), time to peak (*p* < 0.05 at 50 ng/mL, *p* < 0.0001 at 150 ng/mL and 250 ng/mL vs. control) and lag time (*p* < 0.05 at 50 ng/mL, *p* < 0.01 at 150 ng/mL, and *p* < 0.0001 at 250 ng/mL vs. control). 

Edoxaban significantly reduced ETP at the concentration of 250 ng/mL (*p* < 0.05 vs. control) (Figure 1A), peak of thrombin generation at 150 ng/mL (*p* < 0.05 vs. control) and 250 ng/mL (*p* < 0.001 vs. control) (Figure 1B), and at all concentrations reduced time to peak (*p* < 0.05 at 50 ng/mL, *p* < 0.0001 at 150 ng/mL and 250 ng/mL vs. control) and lag time (*p* < 0.05 at 50 ng/mL, *p* < 0.001 at 150 ng/mL and *p* < 0.0001 at 250 ng/mL vs. control) in a concentration-dependent manner.

### 3.2. Platelet Aggregation

**Figure 2:**
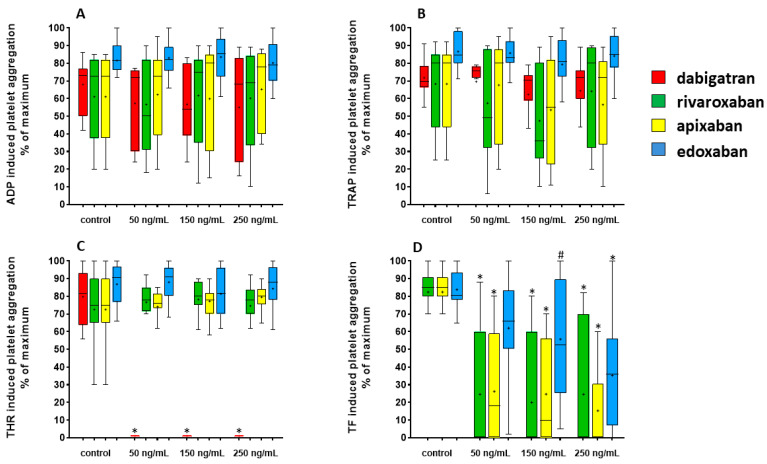
Platelet aggregation induced by different agents after the addition of increasing concentrations of dabigatran, rivaroxaban, apixaban and edoxaban. Box and whiskers Tukey plot reporting the median, 25th and 75th percentile and range for values of platelet aggregation in platelet-rich plasma (optical transmittance, in percent of maximum aggregation) induced by ADP (**A**), thrombin receptor-activating peptide (TRAP) (**B**), gamma-thrombin (THR) (**C**) and tissue factor (TF) (**D**). The asterisk within the bars reports the mean values. * *p* < 0.0001, ^#^ *p* < 0.01, vs. corresponding controls.

Percent values of platelet aggregation induced by ADP, thrombin, TRAP and TF are reported in Table 2 and illustrated in Figure 2. ADP- and TRAP-induced LTA was not modified by the addition of any concentration of NOACs compared with control (Figure 2A,B). Thrombin-induced LTA was completely inhibited by the addition of dabigatran at any concentration (*p* < 0.0001 for all concentrations of drug vs. controls), but not by the other agents (Figure 2C). TF-induced LTA was significantly inhibited by rivaroxaban (*p* < 0.0001 at all concentrations of the drug vs. control), apixaban (*p* < 0.0001 at all concentration of the drug vs. control) and edoxaban (*p* < 0.01 at 150 ng/mL, and *p* < 0.0001 at 250 ng/mL vs. control) (Figure 2D). TF was not used as an agonist to induce platelet aggregation in samples treated with dabigatran, as this was a late addition to the set of aggregating agents during the course of the present study.

### 3.3. Serum TXB_2_ Generation

**Figure 3:**
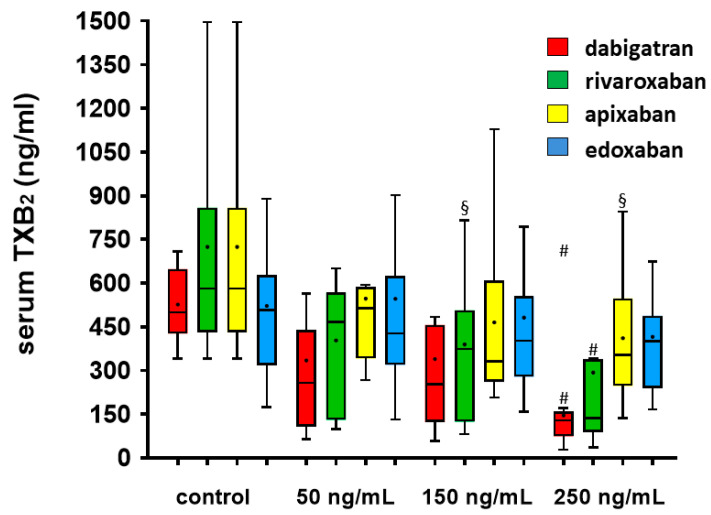
Serum TXB_2_ generation after the addition of increasing concentrations of dabigatran, rivaroxaban, apixaban and edoxaban. Box and whiskers Tukey plot reporting the median, 25th and 75th percentile and range for values of serum TXB_2_. The asterisk within the bars reports the mean values. ^#^ *p* < 0.01, ^§^ *p* < 0.05 vs. corresponding control.

The achieved concentrations of serum TXB_2_ generation is reported in Table 3 and illustrated in Figure 3. The addition of dabigatran in serum at the concentration of 250 ng/mL significantly reduced the generation of TXB_2_ compared with control (*p* < 0.01). Rivaroxaban determined a concentration-dependent reduction in serum TXB_2_ generation, particularly at concentrations of 150 ng/mL (*p* < 0.05) and 250 ng/mL (*p* < 0.01). Apixaban and edoxaban significantly reduced serum TXB_2_ generation at the concentration of 250 ng/mL (*p* < 0.05 and *p* < 0.01, respectively). 

### 3.4. Flow Cytometry

**Figure 4:**
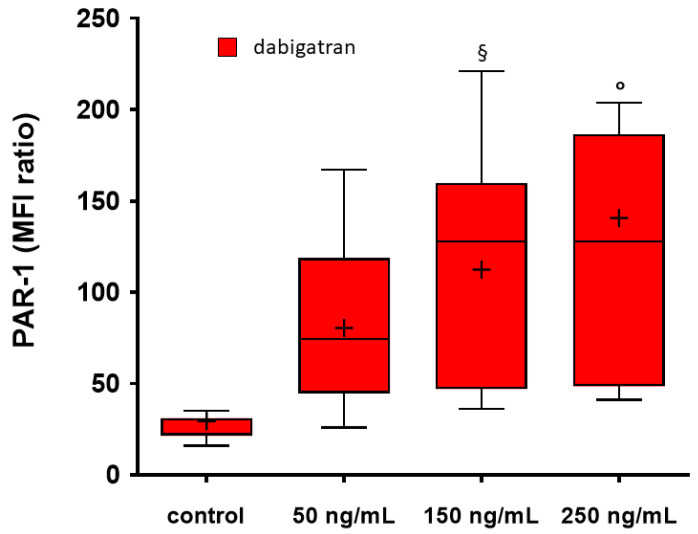
Expression of PAR-1 on platelet surface using flow cytometry without and with the addition of increasing concentrations of dabigatran. Box and whiskers Tukey plot reporting the median, 25th and 75th percentile and range for values of mean fluorescence intensity (MFI) ratio. The «+» within the bars indicate the mean values. ° *p* < 0.001 vs. control; ^§^ *p* < 0.05 vs. control.

The MFI ratio values indicating the expression of PAR-1 and P-selectin on the platelet surface from flow cytometry are reported in Table 4. The proportion of activated platelet over the total according to the expression of P-selectin was not modified by the addition of any of the tested drugs. We observed a concentration-dependent increase in PAR-1 expression with the addition of dabigatran, particularly at concentrations of 150 ng/mL (*p* < 0.05 vs. control) and 250 ng/mL (*p* < 0.001 vs. control) (Figure 4). No significant change in PAR-1 expression was observed with the addition of FXa inhibitors.

## 4. Discussion

In this ex vivo study, we assessed the effect on platelet function of the four currently available NOACs: the thrombin inhibitor dabigatran, and the FXa inhibitors rivaroxaban, apixaban and edoxaban. We tested increasing concentrations of 50, 150 and 250 ng/mL of the drugs, reflecting the similar range of concentrations measured in patients’ plasma during chronic therapy with all these NOACs [22,23,24,25]. We observed the following:

- All NOACs reduced thrombin generation measured by CAT; ETP is the parameter more consistently affected by the addition of NOACs at different concentrations;
- Platelet aggregation induced by ADP and TRAP was not affected by the addition of any NOACs; conversely, platelet aggregation induced by thrombin was significantly reduced by the addition of dabigatran, and TF-induced platelet aggregation is inhibited by FXa inhibitors;
- Serum TX generation was reduced by the addition of all NOACs;
- The expression of PAR-1 on the platelet surface, as evaluated by flow cytometry, was increased by the addition of dabigatran in a concentration-dependent manner, without an enhancement of platelet activation.

All three direct FXa inhibitors and the direct thrombin inhibitor dabigatran have been shown to have a superior net clinical benefit compared with vitamin K antagonists for the treatment of venous thromboembolic events and for the prevention of stroke in patients with atrial fibrillation. However, landmark trials have also reported differences in comparison with warfarin regarding the frequency of atherothrombotic and ischemic events, raising concern, even in the absence of direct clinical comparisons, that NOACs are not all equal and not fully interchangeable. Accordingly, in a recent cross-sectional study analyzing the coagulation response to dabigatran, argatroban, rivaroxaban and apixaban, added at a concentration of 1 μM in blood from 50 healthy donors, the authors underlined some differences between NOACs and indicated that rivaroxaban was the most active according to PT/INR, that BMI had an apparent impact on anticoagulant effects, and that glucose and lipid levels had a direct influence on the effect of anticoagulant drugs ex vivo [33]. 

An initial concern with dabigatran in patients with atrial fibrillation, showing higher rates of myocardial infarction compared with warfarin-treated patients, has been partially, but not totally dissipated [34,35,36]. To explain a possibly higher risk of myocardial infarction in dabigatran-treated compared with warfarin-treated patients, hypotheses have been raised for an augmented GP Ibα signaling downstream of von Willebrand factor binding [37], or enhanced platelet reactivity and increased platelet surface expression of PAR-1 and PAR-4 [18]. On the other hand, FXa-inhibitor landmark trials have reported numerically fewer myocardial infarctions compared with warfarin [38,39,40,41], and basic science studies have investigated potential mechanisms for this apparently protective effect, with discrepant results [5,20,42]. Because platelets play an important role in the pathogenesis of arterial ischemic events and in mechanisms of maintenance of normal homeostasis, investigation on ex vivo effects of NOACs on platelet function and on global hemostasis may help understand the differences between actions among these drugs.

As expected, all NOACs determined a reduced activation of coagulation, as measured by a global coagulation test such as thrombin generation, a test reporting on the plasma potential to generate thrombin. Although some discrepancies between drugs were observed in the various thrombin generation parameters here investigated, the net amount of thrombin generated from plasma appears to be reduced by all drugs, in agreement with previous reports [4,5,6,7]. In particular, we observed that dabigatran inhibited ETP, but did not significantly affect peak thrombin generation, time to peak or lag time. On the other hand, FXa inhibitors modified all thrombin generation parameters: specifically, rivaroxaban and apixaban did so at all concentrations tested, while edoxaban reduced ETP only at the highest concentration (250 ng/mL) and the other parameters also at lower concentrations. These findings are consistent with a recent report showing that dabigatran inhibits thrombin generation in a manner different from FXa inhibitors: in patients treated with NOACs, dabigatran reduced ETP, weakly decreased thrombin peak and did not influence the velocity index, while FXa inhibitors markedly reduced the thrombin peak and velocity index, but had little (in the case of rivaroxaban) or no effect on ETP [43,44]. The authors of these studies proposed that such differences depend on the greater profibrinolytic activity—evaluated by the clot lysis time after exposure to exogenous tissue plasminogen activator—of dabigatran compared with the FXa inhibitors. 

Our data overall demonstrate an inhibitory effect of NOACs on platelet function. Platelet aggregation was affected by the NOACs according to their mechanism of action: the direct thrombin inhibitor dabigatran completely suppressed platelet aggregation induced by γ-thrombin, while FXa inhibitors all reduced platelet aggregation induced by TF, due to the inhibition of thrombin generation as a consequence of blocking the upstream FXa protease, consistent with previous studies [8,13]. In our ex vivo model, NOACs had no effect on platelet aggregation induced by TRAP and ADP: this can be explained because TRAP directly binds thrombin receptors on the platelet surface, bypassing the drug’s inhibitory effect on thrombin generation, and because ADP activates platelets through mechanisms not affected by the inhibition of thrombin or FXa. Otherwise, previous in vivo studies demonstrated that TRAP-induced platelet aggregation was inhibited in patients treated with dabigatran [15], apixaban and rivaroxaban [19,20,21], while ADP-induced platelet reactivity was not influenced [16]. The different behavior of TRAP-induced aggregation could be explained by the different experimental conditions, including the limited time of exposure to the drugs in our ex vivo model.

Serum TXB_2_ generation, reflecting the maximal biosynthetic capacity of blood platelets to generate TXA_2_ in response to endogenously formed thrombin, was significantly reduced after the addition of dabigatran at the highest concentration of 250 ng/mL, nominally consistent with the inhibition of thrombin by this drug, but conflicting with some in vivo evidences [45,46]. However, apixaban and edoxaban at the same concentration, and rivaroxaban even more substantially already at 150 ng/mL, also inhibited serum TXB_2_ generation. These data are consistent with those of a study conducted on patients with atrial fibrillation, showing a significant reduction in 11-dehydro-TXB_2_, the main urinary metabolite of TXB_2_, in patients treated with rivaroxaban and apixaban compared with warfarin [42]; that finding was related to the shedding of soluble GPVI, letting the authors postulate a further mechanism of antiplatelet effect by FXa inhibition. 

The results of flow cytometry experiments documented a concentration-dependent increase in PAR-1 expression on the platelet surface induced by dabigatran, but not by FXa inhibitors, in the absence of platelet activation, as demonstrated by the unchanged expression of P-selectin. This increased density of thrombin receptors on the platelet surface was also observed in patients on long-term treatment with dabigatran [16,18], and can be explained by an inhibitory effect of dabigatran on thrombin-induced PAR-1 cleavage, activation, internalization and β-arrestin recruitment observed in some in vitro studies [17], though little is known about the functional consequences of these “induced” receptors. The augmented density of thrombin receptors may theoretically have a clinical impact during anticoagulant therapy with dabigatran, particularly in conditions of enhanced thrombin generation or when dabigatran concentrations are reduced due to drug interaction or poor patient adherence to therapy. 

Ours is the most comprehensive ex vivo study of the effects of the four currently available NOACs on platelet function, showing similarities but also differences in the pharmacodynamic characteristics of these drugs. None of the previous studies has conducted such comprehensive investigations of platelet function parameters with all the currently available NOACs. This study is now being complemented by parallel in vivo investigations, which will be the subject of an independent report. Together, they will help explaining similarities and differences in clinical outcomes with the currently available NOACs. 

This study also has, however, limitations: an important one is a thorough understanding of the clinical relevance of the sometimes subtle differences found among NOACs, as here reported. Another limitation is that this study was conducted in two distinct laboratories and at different times for the various drugs and this could have affected the reproducibility of some results and the (minor) incompleteness of the entire set of tests during the study, with TF-induced platelet aggregation not performed in the initial blood samples treated with dabigatran. 

## 5. Conclusions

In conclusion, this study shows that treatment with these NOACs is associated with measurable ex vivo changes in platelet function, arguing for antiplatelet effects beyond the well-known anticoagulant activities of these drugs. Among the NOACs, dabigatran appears to be associated with a concentration-dependent increase in PAR-1 expression, suggesting the need for attention to the modulation of platelet-dependent atherothrombotic risk.

## Figures and Tables

**Table 1:** Thrombin generation parameters without and with the addition of increasing concentrations of dabigatran, rivaroxaban, apixaban and edoxaban.

	**Dabigatran**				**Rivaroxaban**			
	**Control**	**50 ng/mL**	**150 ng/mL**	**250 ng/mL**	**Control**	**50 ng/mL**	**150 ng/mL**	**250 ng/mL**
	median (IQR)	median (IQR)	median (IQR)	median (IQR)	median (IQR)	median (IQR)	median (IQR)	median (IQR)
ETP (nM × min)	1918 (1202–2591)	965 (600–2048) ^§^	808 (539 –1516) ^#^	642 (406–1118) *	1919 (1203–2591)	1047 (773–1725) ^§^	806 (647–1408) °	765 (555–1342) *
Peak (nM)	240 (147–285)	133 (100–298)	200 (130–271)	181 (68–216)	227 (128–278)	67 (49–88) *	52 (36–88) *	49 (32–76) *
Time to peak (min)	6.2 (5.6–7.7)	4.7 (3.7–7.0)	4.7 (3.3–11.4)	4.0 (3.2–12.9)	6.2 (5.6–7.7)	13.0 (7.8–17.2) °	12.5 (9.8–20.9) *	15.9 (13.2–18.6) *
Lag time (min)	1.33 (0.7–2.4)	2.3 (1.5–3.2)	2.3 (0.7–6.9)	2.0 (1.0–6.8)	1.3 (0.7–2.4)	3.0 (1.6–4.8) ^§^	4.5 (2.9–6.5) *	5.8 (4.4–7.6) *
	**Apixaban**				**Edoxaban**			
	**Control**	**50 ng/mL**	**150 ng/mL**	**250 ng/mL**	**Control**	**50 ng/mL**	**150 ng/mL**	**250 ng/mL**
	median (IQR)	median (IQR)	median (IQR)	median (IQR)	median (IQR)	median (IQR)	median (IQR)	median (IQR)
ETP (nM × min)	1918 (1202–2591)	1158 (780–1934) ^§^	1100 (697–1482) ^#^	949 (520–1295) °	1218 (975–1794)	1118 (853–1720)	938 (805–1464)	819 (734–1039) ^§^
Peak (nM)	227 (128–278)	77 (50–136) *	62 (40–88) *	49 (38–70) *	101 (85–142)	97 (63–182)	62 (53–96) ^§^	50 (39–69) °
Time to peak (min)	6.2 (5.6–7.7)	10 (6.6–14.3) ^§^	14 (9.8–20.6) *	15 (12–19) *	11 (9–13)	14 (12–17) ^§^	18 (15–20) *	19 (17–22) *
Lag time (min)	1.3 (0.7–2.4)	2.7 (1.7–4.5) ^§^	3.3 (1.6–6.0) ^#^	4.3 (3.6–6.8) *	5.3 (4.9–7.8)	8.2 (6.0–10.2) ^§^	11 (7.4–13.3) °	12 (10.0–13.9) *

ETP = endogenous thrombin potential; IQR = interquartile range. * *p* < 0.0001 vs. control; ° *p* < 0.001 vs. control; ^#^ *p* < 0.01; ^§^ *p* < 0.05 vs. control.

**Table 2:** Platelet aggregation induced by different agents without and with the addition of increasing concentrations of dabigatran, rivaroxaban, apixaban and edoxaban.

**LTA (% of Maximum)**	**Dabigatran**				**Rivaroxaban**			
**Inducing Agent**	**Control**	**50 ng/mL**	**150 ng/mL**	**250 ng/mL**	**Control**	**50 ng/mL**	**150 ng/mL**	**250 ng/mL**
	median (IQR)	median (IQR)	median (IQR)	median (IQR)	median (IQR)	median (IQR)	median (IQR)	median (IQR)
ADP	73 (50−77)	72 (30−76)	54 (39−80)	68 (24−83)	73 (38−82)	50 (31−82)	75 (35−82)	69 (34−84)
TRAP	70 (66−79)	76 (72−78)	71 (59−73)	72 (60−76)	80 (44−85)	49 (32−88)	36 (26−81)	80 (32−89)
Thrombin	82 (64−93)	0 (0−0) *	0 (0−0) *	0 (0−0) *	75 (65−90)	78 (72−85)	80 (75−88)	78 (70−84)
Tissue Factor	NA	NA	NA	NA	85 (80−91)	0 (0−60) *	0 (0−60) *	0 (0−70) *
**LTA (% of maximum)**	**Apixaban**				**Edoxaban**			
**Inducing Agent**	**Control**	**50 ng/mL**	**150 ng/mL**	**250 ng/mL**	**Control**	**50 ng/mL**	**150 ng/mL**	**250 ng/mL**
	median (IQR)	median (IQR)	median (IQR)	median (IQR)	median (IQR)	median (IQR)	median (IQR)	median (IQR)
ADP	73 (38−82)	73 (39−82)	80 (30−85)	78 (40−86)	76 (82−90)	82 (76−89)	86 (73−94)	79 (70−91)
TRAP	80 (44−85)	80 (34−88)	55 (23−82)	72 (34−81)	85 (80−98)	83 (80−93)	81 (73−93)	85 (78−96)
Thrombin	75 (65−90)	76 (73−82)	78 (70−82)	80 (76−84)	91 (77−97)	91 (80−96)	82 (70−96)	88 (78−97)
Tissue Factor	85 (80−91)	18 (0−59) *	10 (0−56) *	0 (0−31) *	81 (78−94)	66 (50−83)	53 (25−90) ^#^	36 (7−56) *

ADP = adenosine diphosphate; IQR = interquartile range; LTA = light transmission aggregometry; THR = thrombin; TF = tissue factor; TRAP = thrombin receptor activating peptide. * *p* < 0.0001 vs. control; ^#^ *p* = 0.01 vs. control.

**Table 3:** Serum TXB_2_ generation without and with the addition of increasing concentrations of dabigatran, rivaroxaban, apixaban and edoxaban.

	**Dabigatran**				**Rivaroxaban**			
	**Control**	**50 ng/mL**	**150 ng/mL**	**250 ng/mL**	**Control**	**50 ng/mL**	**150 ng/mL**	**250 ng/mL**
	median (IQR)	median (IQR)	median (IQR)	median (IQR)	median (IQR)	median (IQR)	median (IQR)	median (IQR)
Serum TXB_2_ (ng/mL)	649 (500−708)	439 (258−1098)	457 (253−1070)	158 (129−470) ^#^	581 (431−860)	467 (130−568)	373 (124−507) ^§^	137 (88−338) ^#^
	**Apixaban**				**Edoxaban**			
	**Control**	**50 ng/mL**	**150 ng/mL**	**250 ng/mL**	**Control**	**50 ng/mL**	**150 ng/mL**	**250 ng/mL**
	median (IQR)	median (IQR)	median (IQR)	median (IQR)	median (IQR)	median (IQR)	median (IQR)	median (IQR)
Serum TXB_2_ (ng/mL)	581 (431−860)	514 (341−586)	332 (262−608)	354 (246−548) ^§^	507 (318−627)	428 (319−624)	402 (278−555)	400 (239−488) ^#^

IQR = interquartile range; TXB_2_ = thromboxane B_2_. ^#^ *p* < 0.01; ^§^ *p* < 0.05 vs. control.

**Table 4:** PAR-1 and P-selectin expression without and with the addition of increasing concentrations of dabigatran, rivaroxaban, apixaban and edoxaban.

	**Dabigatran**				**Rivaroxaban**			
	**Control**	**50 ng/mL**	**150 ng/mL**	**250 ng/mL**	**Control**	**50 ng/mL**	**150 ng/mL**	**250 ng/mL**
	median (IQR)	median (IQR)	median (IQR)	median (IQR)	median (IQR)	median (IQR)	median (IQR)	median (IQR)
PAR-1 (MFI ratio)	22.1 (21.4−31.1)	74.2 (44.5−118.9)	127.8 (46.9−159.8) ^§^	127.9 (48.4−186.5) ^°^	5.7 (4.6−7.0)	5.7 (4.2−7.4)	5.7 (5.0−8.0)	6.0 (3.9−7.3)
CD62+/CD41+ (MFI %)	0.6 (0.1−4.8)	0.8 (0.3−3.9)	0.6 (3.4−1.9)	0.5 (0.1−2.8)	22 (10−27)	16 (10−21)	15 (9−24)	17 (10−31)
	**Apixaban**				**Edoxaban**			
	**Control**	**50 ng/mL**	**150 ng/mL**	**250 ng/mL**	**Control**	**50 ng/mL**	**150 ng/mL**	**250 ng/mL**
	median (IQR)	median (IQR)	median (IQR)	median (IQR)	median (IQR)	median (IQR)	median (IQR)	median (IQR)
PAR-1 (MFI ratio)	5.7 (4.6−7.0)	5.7 (4.5−7.9)	5.7 (4.3−6.8)	5.4 (4.3−6.7)	3.1 (2.0−4.7)	3.6 (2.0−4.8)	3.9 (2.1−4.8)	3.6 (2.1−4.6)
CD62+/CD41+ (MFI %)	22 (10−27)	19 (10−32)	18 (11−26)	16 (13−27)	27 (19−48)	30 (20−49)	32 (22−49)	33 (25−49)

CD41 = platelet GPIIb (IIb integrin); CD62 = P-selectin; IQR = interquartile range; MFI = mean fluorescence intensity; PAR = protease-activated receptors. ° *p* < 0.001 vs. control; ^§^ *p* < 0.05 vs. control.

## Data Availability

The data that support the findings of this study are available from the corresponding author upon reasonable request. Some data may not be made available because of privacy or ethical restrictions.
